# The co-evolved *Helicobacter pylori *and gastric cancer: trinity of bacterial virulence, host susceptibility and lifestyle

**DOI:** 10.1186/1750-9378-2-2

**Published:** 2007-01-04

**Authors:** Yusuf Akhter, Irshad Ahmed, S Manjulata Devi, Niyaz Ahmed

**Affiliations:** 1 Pathogen Evolution Group, Laboratory of Molecular and Cellular Biology, Centre for DNA Fingerprinting and Diagnostics (CDFD), Hyderabad, India; 2 Department of Microbiology, Shri Shivaji College of Arts, Commerce and Science, Akola, India

## Abstract

*Helicobacter pylori *is an important yet unproven etiological agent of gastric cancer. *H. pylori *infection is more prevalent in developing Asian countries like India and it is usually acquired at an early age. It has been two decades since Marshall and Warren (1984) first described curved bacilli in the stomach of ulcer and gastritis patients. This discovery has won them the Nobel Prize recently, but the debate whether *H. pylori *is a pathogen or a commensal organism is still hot. Associations with disease-specific factors remain illusive years after the genome sequences were made available. Cytotoxin-associated antigen A (Cag*A*) and the so-called plasticity region cluster genes are implicated in pathogenesis of the carcinoma of stomach. Another virulence factor VacA whose role is still debatable, has recently been projected in pathology of gastric cancer. Studies of the evolution through genetic variation in *H. pylori *populations have provided a window into the history of human population migrations and a possible co-evolution of this pathogen with its human host. Possible symbiotic relationships were seriously debated since the discovery of this pathogen. The debate has been further intensified as some studies proposed *H. pylori *infection to be beneficial in some humans. In this commentary, we attempt to briefly discuss about *H. pylori *as a human pathogen, and some of the important issues linked to its pathophysiology in different hosts.

'We dance around in a ring and suppose, the secret sits in the middle and knows' – Robert Frost

## Background

Barry J. Marshall and Robin Warren, two Australian researchers who discovered the bacterium *Helicobacter pylori *in 1982 have been awarded Nobel Prize of 2005 in Physiology or Medicine. This 'old fashioned medical detective work' impressed the Nobel Assembly of the Karolinska Institute, to move away from basic research [1,2] and to reward the research that proposes a much controversial bacterial organism as a dangerous pathogen. It was a long-standing dogma in the medical science that stress and lifestyle factors lead to gastritis and peptic ulcer disease. Warren and Marshal rebutted that dogma and made it clear that curved bacilli called *Campylobacter pyloridis *(later named as *Helicobacter pylori*) were the main cause of peptic ulceration, distal gastric adenocarcinoma, and gastric lymphoma [3]. Soon after this, *H. pylori *colonization model became one of the best-studied examples of pathogen evolution and its role in infection biology. This marked also the beginning of a contest on how long *H. pylori *had been colonizing human stomach, promoting the analogy of a symbiotic organism coevolved with its human host.

### *H. pylori *as a marker of human peopling and migration: example of co-evolution

*H. pylori *is presumably co-evolved with its host and therefore, origins and expansion of multiple populations and sub populations of *H. pylori *mirror ancient human migrations. Ancient origins of *H. pylori *in the world and in India are not clear and debatable. It is not clear how different waves of human migrations in different continents contributed to the evolution of strain diversity of *H. pylori*. Our group has recently attempted to address these issues through mapping genetic origins of *H. pylori *of native Peruvians (of Amerindian ancestry) [4] and Indians (Devi *et al*., unpublished data) and their genomic comparison with hundreds of isolates from different geographic regions. For this purpose, genetic identity of strains was dissected by fluorescent amplified fragment length polymorphism (FAFLP) analysis, multilocus sequence typing (MLST) of the housekeeping genes and the sequence analyses of the *bab*B adhesin and *oip*A genes. The whole *cag *pathogenicity-island (*cag*PAI) from these strains was also analyzed using PCR and gene sequencing. In case of South American *H. pylori *populations, it was observed that while European genotype (*hp*-Europe) predominated in native Peruvian strains, approximately 20% of these strains represented a sub-population with an Amerindian ancestry (*hsp-Amerind*). However, all of these strains were shown to harbor a complete, 'western' type *cag*PAI, irrespective of their ancestral affiliation and the motifs surrounding it. This indicated a possible acquisition of *cag*PAI by the *hsp-Amerind *strains from the European strains, during decades of co-colonization. These observations, therefore, suggested presence of ancestral *H. pylori *(*hsp-Amerind*) in Peruvian Amerindians, which possibly managed to survive and compete against the Spanish strains that arrived to the New World about 500 years ago. It was suggested that this might have happened after native Peruvian *H. pylori *strains acquired *cag*PAI sequences, either by new acquisition in *cag*-negative strains or by recombination in *cag *positive Amerindian strains. In case of Indian strains, almost all the isolates analyzed revealed a European ancestry and belonged to MLST genogroup hp-Europe. The *cag*PAI harbored by Indian strains also revealed European features upon PCR based analysis and whole PAI sequencing. These observations suggest that *H. pylori *in India have ancient origins in Europe (Devi *et al*, unpublished data). These results are expected to strengthen speculations related to large-scale replacement of the ancient indigenous people of India by Indo-Aryan nomads, bringing first Neolithic practices and languages from the Fertile Crescent.

### *H. pylori *in gastric diseases

*H. pylori *causes peptic ulceration, gastric adenocarcinoma, and gastric lymphoma. Gastric adenocarcinoma is the second highest cause of cancer deaths worldwide mainly due to high incidence, aggressive disease course, and lack of effective treatment options leading to a death toll of one million per annum worldwide [3]. *H. pylori *is implicated in distal gastric adenocarcinoma, which is more common than the proximal one. *H. pylori *also causes B cell mucosa-associated lymphoid tissue (MALT) lymphoma of the stomach [3] but at the same time negatively associated with more severe forms of reflux esophagitis and its sequelae – Barrett's esophagus and esophageal adenocarcinoma [5,6]. This negative correlation is the main reason that makes *H. pylori *a lesser evil. There has been a recent interest to look if *H. pylori *causes or facilitates human diseases of the gut other than the upper gastrointestinal tract or syndromes like idiopathic thrombocytopenic purpura [5,6], skin diseases, liver diseases, and cardiovascular and cerebrovascular disease. But many of these have been associated more commonly with Helicobacters other than *H. pylori *[7,8].

### Bacterial encoded proinflammatory and carcinogenic factors

Studies reveal that the risk for developing gastric carcinoma was much greater with the *H. pylori *infection [9]. The *cag*A gene of *H. pylori *is the main virulence factor that leads to the development of gastric adenocarcinoma through derangement of cellular architecture and signaling. Presence of a functional *cag*A gene determines the *H. pylori *strain type to be aggressive or mild. The *cag*A-positive strains cause much intense ulceration of stomach or duodenum and are more damaging than the *cag*A-negative ones [10] leading to atrophic gastritis and gastric carcinoma [11,12]. CagA, the effector protein product of *cag*A, is tyrosine phosphorylated by SRC kinases after its secretion on the intestinal mucosal surface [13]. EPIYA motifs in the CagA protein sequence play a critical role in tyrosine phosphorylation, which in turn activates a SHP2 phosphatase to act as a oncoprotein. As SHP2 helps in cell growth and motility, its deregulation by CagA is an important oncogenic mechanism encoded by *H. pylori*. CagA based on sequence variation at the SHP2 binding site, is sub-classified into two main epidemiological types – East-Asian CagA (with stronger SHP2 binding and greater biological activity) and Western CagA (diminished SHP2 binding and milder ulcerative potentials). Strains with multiple CagA tyrosine phosphorylation motifs are more commonly associated with gastric cancer than those with fewer C type motifs [14-16].

Incidence of infection with *H. pylori *carrying biologically more active CagA might explain the high occurrence of gastric carcinoma in some countries such as Japan and Korea. However, other populations with extremely high infection rates, such as Indians have almost negligible incidence of gastric carcinoma [17]. Possible reasons for such strange differences of disease outcome might be explained in the light of differences in genetic susceptibility among host populations, environmental factors such as dietary habits, and strain differences of *H. pylori*.

*H. pylori *has a single copy of the *vac*A gene encoding VacA protein, a secreted 95 kDa peptide. The *vac*A gene varies in the signal sequence (alleles s1a, s1b, s1c, s2) and/or its middle region (alleles m1, m2) among different *H. pylori *populations. The different allotypes of s and m regions determine the extent of cytotoxicity of VacA. Strains with *vac*A genotype s1/m1 are more commonly associated with gastric cancer than the other types [18]. Among other functions, VacA has been shown to induce apoptosis in epithelial cells. Recently, VacA has been proposed to be a potent immunomodulatory toxin, targeting the adapted immune system to suppress local immune responses to prolong the outcome of infection and thus prevent clearance by the host immune system [19]. The VacA has been the subject of intense biochemistry but lacked solid evidences that it is indeed involved in pathogenesis. A recent study argues that VacA has a miniscule role as a virulence factor during cell evasion by *H. pylori*. They showed that the *vac*A null mutant of *H. pylori *was able to evade specific cell lines, as did its wild type [20]. Therefore, the VacA involvement is still part of a debate on its being a true virulence factor and awaits further investigation.

Apart from the cardinal virulence factors CagA and VacA, several other proteins of the *cag*PAI, outer membrane envelope proteins, flagellins, adhesins, neutrophil activating protein (NAP), porins, LPS, urease and some members of the so called plasticity region cluster possibly playing an important role in inflammatory processes.

### Microevolution during colonization: can it be linked to virulence optimization?

It has long been assumed that i) the *H. pylori *virulence factors are stable characteristic amid an otherwise fast evolving and recombining genome and ii) that these factors can be linked to disease progression or outcome, at any time. However, several reports present data against these assumptions. Two subclones of a *H. pylori *strain co-colonized a single patient with variations in *vac*A mid region, rendering one of the two sub-clones non-toxic [21]. The reason for this was clearly the microevolution *via *recombination within the stomach. Our group has previously shown a large deletion in *vac*A gene occurring in one of the two isolates of a common progenitor strain in a French patient, obtained 9 years apart [22]. This was most probably a case of adaptation or evolution *in vivo*. Duplication or deletion of the *cag*A gene has been shown by Aras *et al*., [23] in two isolates existing in one individual and recovered 7 years apart. Kersulyte *et al*, have shown complete deletion of *cag*PAI through recombination [24]. In addition, various genotyping methods applied to two or more *H. pylori *isolates obtained from the same patient revealed similar fingerprints, with minor differences [25,26]. This may be possible due to the fact that two or more isolates recovered from a patient may share an ancestral relationship with a founder strain but have undergone independent genomic alterations. This phenomenon has been termed as 'microevolution' [25,27]. However, sequence evidence is necessary to confirm the location and extent of microevolution and phenotypic confirmation [16] is required to ascertain if such microevolution leads to alteration or optimization of virulence in response to change in the gastric environment.

### Host genetic factors in *H. pylori *induced carcinoma

Host factors also play an important role in predisposition to *H. pylori *induced diseases and susceptibilities towards severe pathological outcomes. The host factors relevant in *H. pylori *induced diseases mainly include components of gastric secretion system and the immune apparatus. Interestingly, the gastritis and ulcer disease that result from bacterial infection, have distinct clinical profiles and are inversely associated with a high degree of acid secretion, whereas, gastric cancers are associated with low acid secretion due to loss of parietal cell mass [28,29]. In a recent study involving an East Indian population, authors suggested an association between the IL1β gene polymorphisms and *H. pylori*-mediated duodenal ulcer risk. They further observed effects of specific IL1β genotypes on the expression of IL1β mRNA in the gastric mucosa. Their *in vivo *studies were further substantiated, for the first time, by *in vitro *experiments, which represent the opposite homozygous risk genotypes that were observed in duodenal ulcer patients [30]. So this might explain the fact that differences in carcinogenesis risk in people from different geographical areas might reflect differences in their genetic make up.

### The developing country enigma: Indians, diet and predisposition to gastric cancer?

What is enigmatic about the gastric cancer scenario in India? The answer is not simple. This country has a high prevalence of *H. pylori *infections and a low risk of gastric cancer in contrast to some of the developed countries with a low *H. pylori *colonization rate like China and Japan. India is known for a very high incidence of *H. pylori *infection [31,32]. Biologically inactive CagA could be a contributory factor in low prevalence of gastric ulcers and cancer in India. However, phenotyping studies based on *in vitro *assessment of CagA function in Indian isolates have not been done. In our opinion it will be inappropriate to implicate CagA functionality alone. The spectrum and outcome of pathology in *H. pylori *infection is intricately governed by all the three factors – virulence, host genetics and the environment. It appears that the environment of stomach (acidity, buffering and mucus content) governed by lifestyle factors (diet, food habits, alcoholism, oral hygiene, water hygiene, personal hygiene, proximity with farming communities and animals) and the genetic determinants of susceptibility are chief drivers of the pathological outcome. Although poverty-associated factors (overcrowding, poor sanitation, lower socioeconomic status, compromised water hygiene etc.) in countries like India facilitate high frequency of *H. pylori *colonization, rapid re-colonization post eradication and lower age of acquisition [33]; a surprising fact is that such areas are at lowest risk of developing gastric cancer [34]. Correlation between *H. pylori *infection and gastric cancer has so far been unsuccessful in India [35]. A recent study from India involving 279 patients with gastric neoplasms failed to show a higher prevalence of *H. pylori *infection in patients with gastric neoplasms as compared to the controls (101 non-ulcer dyspepsia and 355 healthy subjects) [36]. These observations challenge the versatility of simplified models of gastric carcinogenesis based on *H. pylori *infection. We believe that in Indian context, diet as a major environmental factor governs the dynamics of gastric cancer demography mainly by regulating physiological integrity of gastric mucosal niches. And that is where; the dietary practices and lifestyle factors become important in the context of progression from gastritis to gastric cancer. Diets low in vegetables, fibers and fruits and high in salt-preserved foods or salt-processed meat increase the risk of stomach cancer [37].

Accordingly, in such situations there seems to be a difference in the distribution frequency of gastric cancer incidence. The southern and eastern parts of India have higher frequency of gastric cancer than rest of the country [38]. Rice is the staple food in south, whereas fish, meat, spices and salts are the main food items in eastern part [37-39]. Contrastingly, the large vegetarian population in northern India is at lower risk of gastric cancer. But the times are changing; rapid flourish of post globalization corporate culture brought fast foods, germ-free bottled water, pasteurized milk and preserved meat items to the present day lifestyle in big Indian cities. However, it will be too early to link it with rising gastric cancer incidences in cities in India [39].

Conversely, low to negligible incidence of gastric cancer as recorded for rural areas in India by the national cancer registry [39] leads us to speculate why rural communities have distinct advantages in terms of less damages from *H. pylori *infection. It needs to be investigated if these advantages are due to their diet based on fresh farm produce and their 'friendship' with the so-called "old friends", the group of bacteria that might be maintaining levels of regulatory immune cell populations and have been intricately associated during most of the mammalian evolution.

## Competing interests

The author(s) declare that they have no competing interests.
